# Could the Anatomic Variants of the Superior Thalamic Vein (STV) Be Considered a Possible Landmark for Target Identification in Magnetic-Resonance-Guided Focused Ultrasound Procedures? A Pilot Study Using Susceptibility Weighted Imaging Sequences

**DOI:** 10.3390/diagnostics14131409

**Published:** 2024-07-02

**Authors:** Simona Cammaroto, Giuseppe Acri, Valentina Hartwig, Rosa Morabito, Annalisa Militi, Chiara Smorto, Augusto Ielo, Lilla Bonanno, Carmelo Anfuso, Angelo Quartarone

**Affiliations:** 1 IRCCS Centro Neurolesi “Bonino Pulejo”, 98124 Messina, Italy; simona.cammaroto@irccsme.it (S.C.); rosa.morabito@irccsme.it (R.M.); annalisa.militi@irccsme.it (A.M.); chiara.smorto@irccsme.it (C.S.); augusto.ielo@irccsme.it (A.I.); lilla.bonanno@irccsme.it (L.B.); carmelo.anfuso@irccsme.it (C.A.); angelo.quartarone@irccsme.it (A.Q.); 2 Dipartimento di Scienze Biomediche, Odontoiatriche e Delle Immagini Morfologiche e Funzionali, Università degli Studi di Messina, c/o A.O.U. Policlinico “G. Martino”, 98125 Messina, Italy; 3 Institute of Clinical Physiology, National Research Council—CNR, 56124 Pisa, Italy

**Keywords:** MRgFUS, SWI sequences, VIM localization, STV variants

## Abstract

During magnetic-resonance-guided focused ultrasound ablation of the ventral intermediate thalamic nucleus (VIM) for essential tremor (ET) and Parkinson’s disease (PD), targeting is generally performed using a standard atlas-based stereotactic approach. The purpose of our work is to evaluate the anatomic variations in the venous vasculature of the thalamus in patients treated with MRgFUS, as a possible landmark for targeting. We retrospectively evaluated the relationship between the obtained thalamotomy lesion and the ipsilateral superior thalamic vein (STV). A total of 36 patients (25 ET and 11 PD) who underwent MRgFUS treatment were evaluated, and the STV was studied with susceptibility weighted imaging (SWI) sequences. Based on the axial SWI images, the distance between the STV and the center of the lesion at the presumed site of the VIM was measured in follow-up MRI images one month after treatment. Statistical analysis shows that there is a correlation between the STV and the presumed site of the VIM. The STV visible in SWI could be used as an additional, real-time, and patient-specific anatomical landmark for VIM identification during MR examination and just before and during FUS treatment.

## 1. Introduction

Magnetic resonance-guided focused ultrasound (MRgFUS) is a hybrid technique that uses magnetic resonance (MR) to obtain morphological information and ultrasound (US) for tissue ablation [1]. In particular, MR is a non-invasive diagnostic method that does not involve ionizing radiation [2], but it uses static and radiofrequency magnetic fields to obtain anatomic information about the organs inside the human body [3]. US transducers are coupled with an MR scanner, either in the head coil or in the table, to generate energy that causes a significant increase in the temperature of the targeted tissues [4]. This hybrid technique is used in various medical applications, from breast cancer to uterine fibroids [5,6,7]. In recent years, MRgFUS has been used to treat essential tremor (ET) and Parkinson’s disease (PD) [8,9,10]. ET is the neurological cause of postural or action tremor. The 2018 consensus statement on the classification of tremors had defined ET as a syndrome of bilateral upper limb action tremor in the absence of other neurological symptoms, present for at least 3 years, with or without tremor at additional sites [11]. The 2018 consensus statement consistently added the essential tremor plus (ETP) classification to identify those patients with subtle neurological signs in addition to postural tremor [AA]. There is an ongoing debate about the differentiation of ET disease into these two classes [12,13]. Although ET is generally considered benign, it can often lead to embarrassment and, in a small percentage of patients, serious disability [14]. The symptoms of PD are typically progressive and potentially disabling, with tremor being the initial symptom [15]. PD is a neurodegenerative disorder characterized by progressive asymmetric slowness of movement, rigidity, tremor, gait disturbance, and a wide range of non-motor symptoms. The etiology of Parkinson’s disease is multifactorial, involving both genetic and environmental risk factors [16]. The prevalence of Parkinson’s disease is increasing worldwide, and it is considered the second most common neurodegenerative disorder [17]. Current treatment strategies are predominantly centered on symptom management. Disease-modifying treatments are crucially needed to mitigate the development of the most debilitating refractory symptoms, including gait and balance difficulties, cognitive impairment and dementia, as well as speech and swallowing impairments [18].

Today, deep brain stimulation (DBS) is being proposed as neurosurgical intervention for the treatment of ET and PD [19]. However, this procedure is invasive, as it requires the insertion of intracerebral electrodes and an implanted pulse generator inside the skull [20]. In this context, MRgFUS is being explored as a possible non-invasive treatment for ET and PD. MRgFUS treatments involve the ablation of a small brain target. This target is represented by the ventral intermediate (VIM) thalamic nucleus. The standard initial targeting method employs an approximate, atlas-based stereotactic approach derived from the VIM [21,22] in the Talairach brain with limited individual-specific adjustment based on the proportional distance between the anterior commissure (AC) and posterior commissure (PC) and the distance from the ventricular wall. In particular, in the classical method of targeting the VIM, a straight line is first drawn and measured on an axial plane connecting the ventricular borders of the AC and PC. The y coordinate is positioned at one-quarter the length of the intercommissural line (ICL) in front of the PC, while the x coordinate is placed 14 mm to the side of this point. The z coordinate is identical to the AC–PC plane [23]. However, this methodology does not consider the individual anatomy of each patient. Therefore, an alternative approach was proposed. In reference [24], a new targeting method was developed based on a highly sensitive and robust MR sequence for imaging gray matter–white matter contrast to identify the VIM. This MR sequence is characterized by a shorter inversion time compared to the standard one and a T2-weighted sequence that can highlight subcortical gray matter structures such as the nuclei of the thalamus.

The authors obtained the VIM target using both manual and automatic segmentation techniques. The automatic segmentation technique used was thalamus optimized multiatlas segmentation (THOMAS). Recent studies have investigated the personalized targeting of the ventral intermediate nucleus (VIM) through the use of connectivity based on diffusion tensor imaging (DTI). These approaches identify the VIM based on its known connection with the dentato-rubro-thalamic tract [25,26] or by segmenting the thalamus based on its connectivity with the cerebral cortex [27,28]. The direct tractographic approach is currently a valuable aid in assessing the target space position. However, technical difficulties are associated with DTI for the thalamus [29]. The aim of our study is to propose a different approach to VIM identification. The methodology is based on the relationship between the VIM and the ipsilateral superior thalamic vein (STV).

In particular, the STV and its respective anatomical variants were identified, and 78 patients were classified according to the Dorfer et al. scheme, as reported in their “Proposed Classification (2018)” [30]. The aforementioned study establishes seven sub-groups of variants: Type 1, which involves drainage into the internal cerebral vein (ICV) and can be divided into Type 1A and Type 1B, in which the STV drains into the anterior portion of the ICV and into the posterior portion of the ICV, respectively; Type 2, where the STV drains into the Rosenthal vein; Type 3, in which the STV drains in the atrial vein and can also be divided into Type 3 A and Type 3 B, in which drainage occurs in the medial atrial vein and in the lateral atrial vein, respectively; Type 4, in which the STV drains into the Galeno vein; and the last and less common variant is represented by the double STV, which combines the variants Type 1B and Type 2.

Specifically, using susceptibility weighted imaging (SWI) sequences obtained with 3T MRI, STV variants were identified, and the Euclidean distance between STV and the VIM was measured. The STV localization, which is variable for each patient, could be used as a possible anatomical reference for VIM identification.

## 2. Materials and Methods

In this retrospective study, 36 patients were enrolled to investigate the ability of our proposed methodology in VIM identification, and we compared the obtained results with VIM identification made by using the standard atlas-based stereotactic approach and with the position of the thalamotomy made by the focused ultrasound embedded on the MR device.

The patients were retrieved according to the following inclusion criteria: a. successful treatment (tremor improvement at the end of the treatment, defined as >50% Clinical Rate Score for Tremor (CRST) reduction with respect to baseline) and b. availability of pre- and post-3 T MR images and of all procedural reports (description of intraprocedural sonication parameters, target coordinates, clinical events).

The patients underwent unilateral MRgFUS VIM ablation in the period between January 2021 and June 2022 at our IRCCS Centro Neurolesi Bonino-Pulejo of Messina, Italy. In Table 1, the demographic and clinical features are reported.

All procedures in this study were performed in accordance with the ethical standards of the 1964 Declaration of Helsinki and its later amendments and approved by the Institutional Review Board of the IRCCS Bonino Pulejo (CE n.38/2021). Informed consent was obtained from all individual participants included in this study.

All patients received unilateral MRgFUS treatment (ExAblate 4000, InSightec, Haifa, Israel) of the VIM nucleus; they were ablated in the contralateral thalamus corresponding to the dominant hand.

Pretreatment 3 T MR images were acquired on a Philips Achieva 3 T dStream scanner (Philips Healthcare, Best, The Netherlands), using a 32-channel head coil. The sequences used were 3D T1-weighted Magnetization Prepared Rapid Gradient Echo (MP-RAGE), 3D-Fluid-Attenuated Inversion Recovery (3D-FLAIR), 2D coronal and axial T2-weighted Fast Spin Echo (FSE), and axial susceptibility weighted imaging (SWI). The scan parameters for each sequence used are reported in Table 2.

Based on the pretreatment SWI sequence, the STV and its respective anatomical variants were identified following the Dorfer scheme [30].

The second step was the identification of STV; in particular, the part where the deepest thalamic vein converges to become STV and its spatial coordinates were recorded by using Insightec software, version 7.33.

After 4 weeks from MRgFUS treatment, the patients underwent the 3 T MR exam, always using the Philips Achieva 3 T dStream scanner. The 3 T post-treatment images were used to detect and characterize ablation features. In particular, the geometric center of the thalamotomy lesion focus was evaluated, and the spatial coordinates were measured and recorded. The lesion center was measured at its maximum extent on a two-dimensional plane, including the three zones according to Wintermark [31].

**Figure 1:**
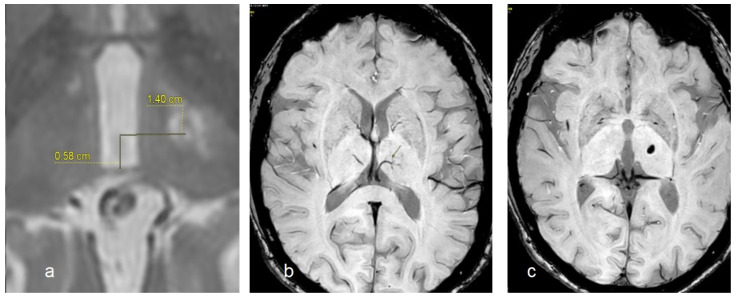
Target identification by using stereotactic coordinates (**a**), STV visualization (the arrow indicates the vein) by using the SWI sequence (**b**), the real position of target obtained after sonication and evaluated after 4 weeks by using the SWI sequence (**c**).

In Figure 1, we report a comparison of the target identification obtained by using stereotactic coordinates (a), STV identification (b), and the lesion of the target after sonication (c).

The last step consisted of the evaluation of the Euclidean distance computed on pretreatment SWI images and the lesion center distance evaluated on post-MRgFUS treatment images. Two raters, who were neuroradiologist experts with more than 10 years of clinical experience, analyzed the acquired images using always the Insightec software, release 7.33, of the device and performed the measurements.

### Statistical Analysis

For each rater, we calculated the distance between the center of the necrosis and the identified STV. To assess the inter-rater reliability of the measurements involving the spatial coordinates of the points and the distances between them, we employed a multifaceted statistical approach. We applied the root mean square deviation (RMSD) to quantify the average discrepancies in the spatial positioning of the points identified by the two independent raters. The RMSD values provide a measure of precision between raters in locating specific points in space, with lower values indicating higher precision. We used intraclass correlation coefficient (ICC) to evaluate the reliability of the distances between the points selected by the two raters. We considered the ICC2 model for measuring random effects and assuming that the raters were randomly selected from a larger set of expert radiologists. A higher ICC value suggests greater reliability, with values close to 1 indicating near-perfect reliability. Bland–Altman plot analysis was used to graphically describe the agreement between two sets of measurements. Finally, a comparison between the coordinates of the STV and focus points (mean of the two raters’ measurements) was performed with the Mann–Whitney U test. The analysis was conducted using R (version 4.2.3), with the significance threshold set at a *p*-value of <0.05.

## 3. Results

**Figure 2:**
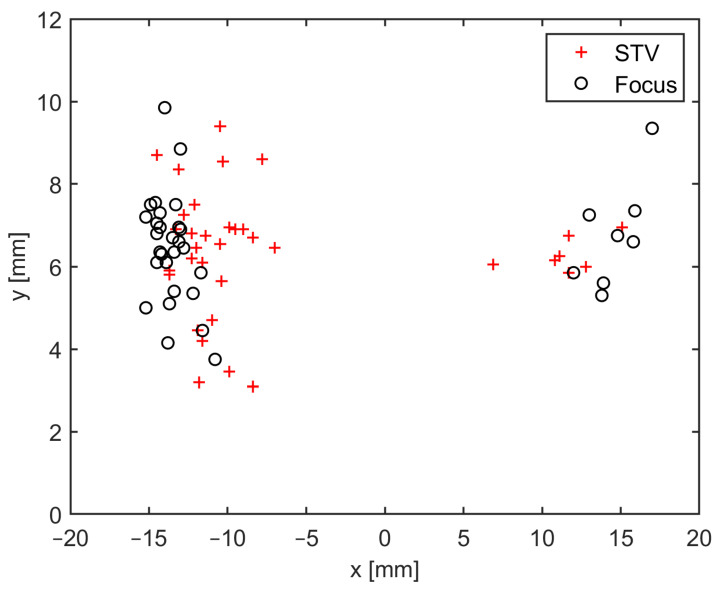
STV and focus coordinates (mean of the two raters’ measurements) on xy plane (axial plane). STV = superior thalamic vein.

The scatter plot in Figure 2 shows the coordinates of the STV and focus (mean of the two raters’ measurements) on the axial plane.

Table 3 shows the average of the coordinate values of the STV and focus (mean of the two raters’ measures) on the axial plane, together with the Euclidean distance between the STV and focus, for the entire population and for the three most representative anatomic variants 1A, 1B, and 3A.

**Figure 3:**
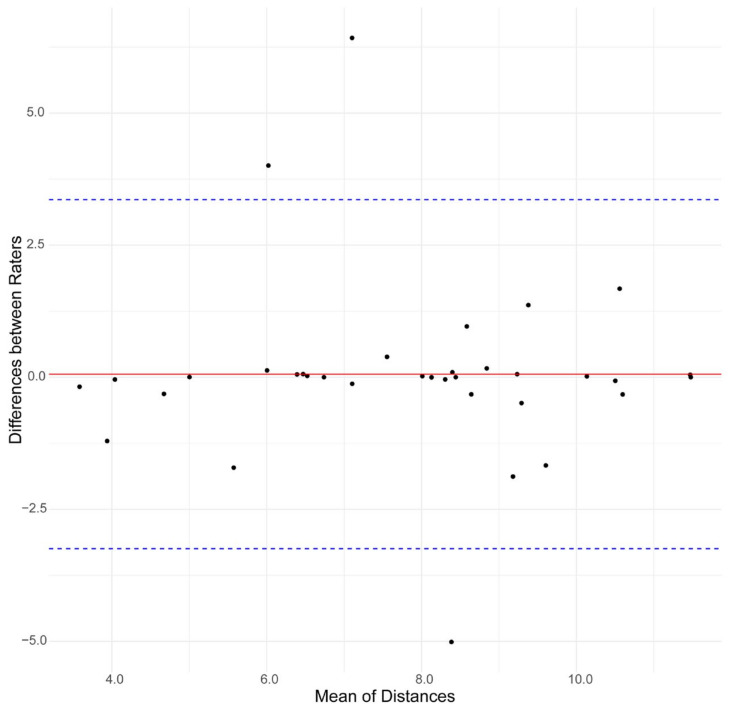
Bland–Altman plot. Agreement between the two raters’ distance measurements. Distance between actual target and STV.

The RMSD analysis revealed an average of 3.45 for the STV and 3.88 mm for the actual target. The ICC analysis highlighted a value of 0.72 (*p* < 0.001) for the actual target to the STV-measured distances. The Bland–Altman plot analysis (Figure 3) visually confirmed the agreement between the two raters’ distance measurements.

The mean distance from the actual target and the STV was 3.15 ± 1.77 mm, considering all the anatomic variants in the population.

**Figure 4:**
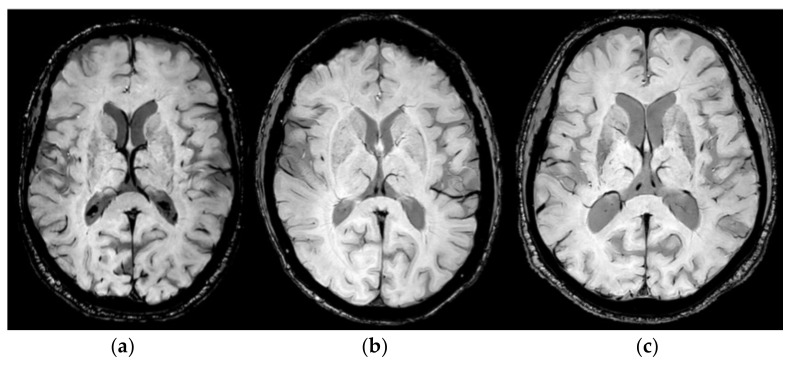
Example of STV variants obtained by using SWI MR sequences for three different patients enrolled in this study. (**a**) Related to Type 1 A variant; in (**b**), Type 1 B variant is shown; and, in (**c**), Type 3 A variant is depicted.

In Figure 4, examples of the detected STV variants from three different patients and those obtained by using SWI sequences are reported. In Figure 4a, it is possible to observe the Type 1A variant in which the STV drains into the anterior portion of the ICV; in Figure 4b, the Type 1 B variant, in which the STV drains into the posterior portion of the ICV, is depicted; and in Figure 4c, the variant Type 3 A is shown, in which drainage occurs in the medial atrial vein.

**Figure 5:**
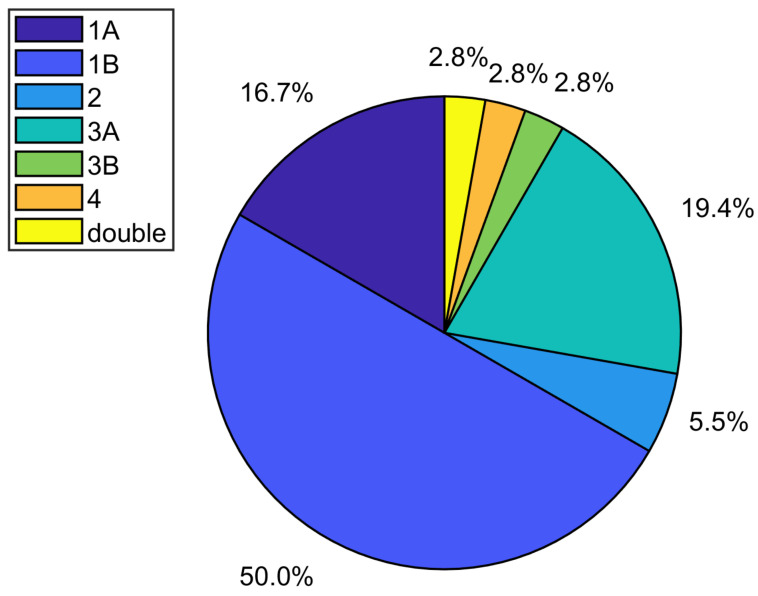
Percentage of STV variants visualized in the patients enrolled in this study.

By using the Dorfer STV classification [30], in this retrospective study, we have visualized the anatomic variants that came closest to the focus, and, in Figure 5, we report the percentage of the variants found in our population.

## 4. Discussion

In order for the MRgFUS treatments to have a good therapeutic impact, it is crucial to precisely localize the target (VIM). For this reason, the identification and evaluation of the VIM is a matter of debate in MRgFUS treatments. The conventional approach uses atlas-based stereotactic coordinates, but this approach does not consider the actual anatomy of the patient. Alternatively, tractography-based methods are being developed. In this paper, we propose an alternative landmark in VIM identification based on the MR SWI sequence, consisting of the localization of the STV and its anatomic variations.

In recent years, magnetic susceptibility sequences have proven to be superior to venographic studies in identifying venous structures. Additionally, they have the advantage of not requiring a contrast medium and not being contaminated by the arterial system [23].

Magnetic susceptibility weighted sequences utilize the magnetic field’s sensitivity to oxygen in blood vessels, specifically the difference in the magnetic susceptibility between oxygenated (arterial) and deoxygenated (venous) blood.

Specifically, SWI sequences have proven to be useful in identifying venous anatomical variants at the encephalic level. Several groups, as early as 2015, have studied the anatomical variants of the thalamostriate veins and their tributaries [32] or other anatomical variants of the deep venous system in preterm infants, such as Tortora et al. [33].

After evaluating the relevance and reliability of this technique in identifying the aforementioned variants, we applied it to the STV. For this, our study utilized the anatomical variants’ classification of the STV published by Dorfer [30].

In our study, the anatomical variant Type 1B was the most frequent (visible), followed by variants Type 1A and 3A. Variant Type 3B appeared to be the furthest from the focal lesion.

The STV coordinates are close to the focal lesion, in particular along the axial plane when the average distance between the STV and the target is just over 3 mm and even less if the Type 1A and Type 3A variants are considered.

The results indicated a high degree of precision and reliability in the measurements conducted by the two raters, supporting the consistency and accuracy of their assessments.

The Bland–Altman plot demonstrated that the majority of differences between the measurements were included within the limits of agreement, indicating a systematic consistency between the raters, with no significant bias observed.

## 5. Conclusions

The early and accurate detection of VIM is crucial in view of a proper intervention; in particular, during MRgFUS treatments, it can be challenging. In the present pilot study, an alternative anatomic landmark to stereotactic coordinates was presented. Our group retrospectively re-evaluated 36 MRI examinations, performed before and after MRgFUS treatments, and identified the STV as a possible anatomical landmark for VIM identification. The results are very close to the focal lesion, with a main advantage, compared to the stereotaxic coordinates, of considering the real anatomy of each patient. The STV was identified by both raters in all patients enrolled in this study; it does not require the calculation of the AP-PC line and can be localized in real time during the intraoperative step only by using the SWI MR sequence.

Even though the number of enrolled patients was small, the preliminary results are very encouraging. We are fully aware that the low number of patients does not allow us to draw definitive conclusions, and thus our future efforts will be oriented toward expanding the number of patients.

## Figures and Tables

**Table 1:** Demographic and clinical features of the patients enrolled in this study.

	ET (25)	PD (11)
Age (mean ± SD)	68.2 ± 11.7	65.3 ± 8.6
M/F	16 M/9 F	11 M
Left VIM treated	20	7
Right VIM treated	5	4

**Table 2:** RM scan parameters set during examinations.

	MR-RAGE	3D-FLAIR	2D-FSE	SWI
Repetition Time (TR)	8.2 ms	12,000 ms	4100 ms	31 ms
Echo Time (TE)	3.7 ms	140 ms	100 ms	7.2 ms
Slice Thickness (ST)	1 mm	4 mm	2 mm	3 mm
Reconstruction Matrix	512 × 512	512 × 512	512 × 512	512 × 512

**Table 3:** Average values of STV and focus related to all patients enrolled in this study and for the three most representative anatomic variants.

	x (mm)	y (mm)	Euclidean Distance between STV and Focus (mm)
Focus	13.84 ± 1.26	6.51 ± 1.30	-
STV	11.42 ± 2.20 *	6.63 ± 2.21	3.15 ± 1.77
Type 1A (17%)	12.98 ± 1.36	6.73 ± 1.02	2.78 ± 1.28
Type 1B (50%)	10.78 ± 1.73 *	6.42 ± 1.60	3.17 ± 1.77
Type 3A (19%)	12.09 ± 1.77	6.56 ± 1.35	2.53 ± 1.68

* *p* < 0.05 vs. focus.

## Data Availability

The data presented in this study are available on request from the corresponding author.
